# Mandibular Vertical Growth Deficiency After Botulinum-Induced Hypotrophy of Masticatory Closing Muscles in Juvenile Nonhuman Primates

**DOI:** 10.3389/fphys.2019.00496

**Published:** 2019-04-26

**Authors:** Hak-Jin Kim, Hye-Jin Tak, Joo-Won Moon, Sang-Hoon Kang, Seong Taek Kim, Jinquan He, Zhenguo Piao, Sang-Hwy Lee

**Affiliations:** ^1^Department of Oral and Maxillofacial Surgery, College of Dentistry, Yonsei University, Seoul, South Korea; ^2^Oral Science Research Center, College of Dentistry, Yonsei University, Seoul, South Korea; ^3^Department of Oral and Maxillofacial Surgery, National Health Insurance Service, Ilsan Hospital, Goyang, South Korea; ^4^Department of Oral Medicine, College of Dentistry, Yonsei University, Seoul, South Korea; ^5^Department of Oral and Maxillofacial Surgery, Stomatology Hospital of Guangzhou Medical University, Guangzhou, China; ^6^Department of Oral and Maxillofacial Surgery, Oral Science Research Center, College of Dentistry, Yonsei University, Seoul, South Korea

**Keywords:** masticatory muscles, craniofacial, growth, botulinum toxin, computed tomography, mandible, monkey

## Abstract

The purpose of this study was to investigate the relationship between masticatory muscular hypotrophy and mandibular growth in juvenile nonhuman primates (cynolmolgus monkeys, Macaca fasicularis). We hypothesized that botulinum toxin (BTX)-induced neuro-muscular junctional block and its resultant hypotrophy of masticatory muscles would produce mandibular growth disturbances in size and shape. Ten male cynomolgus monkeys were divided into three groups: group I (control; *n* = 3), group II (unilateral BTX; *n* = 4), and group III (bilateral BTX; *n* = 3). The unilateral or bilateral muscular hypotrophy of major masticatory closing muscles was induced by synchronous BTX application to masseter, medial pterygoid, and temporal muscle. Mandibular growth was tracked by linear, angular, area and volume measurements using three-dimensional (3D) computed tomography imaging before BTX treatment and after 3 and 6 months. After unilateral hypotrophy of masticatory muscles in group II, vertical growth deficiency was prominent on the BTX side, with compensatory overgrowth on the control side. The bilateral muscular hypotrophy in group III also showed smaller ramal height and width than that of control (group I) and control side (group II). Moreover, ramal sagittal angles (posterior tilt) increased on the BTX side of both groups II and III, but coronal angles (lateral tilt) did so on the BTX side of group II, resulting in asymmetry. The results confirmed our hypothesis that functional activity of masticatory closing muscles is closely related to mandibular growth in size and shape of juvenile nonhuman primates. In addition, the focused growth disturbances on the ramal height and posterior-lateral tilt suggested the possible role of masticatory closing muscles for ramal vertical and angular growth vector of the mandible.

## Introduction

The bone-muscle relationship may be viewed in terms of structure and function (Cianferotti and Brandi, 2014). Some studies have reported aberrant craniofacial structure after experimental muscular function changes (Boyd et al., 1967; Navarro et al., 1995). However, these growths were also influenced by the secondary effects of scar tissues and their subsequent contractures. This issue can be avoided by the introduction of botulinum toxin (BTX), which blocks the nerve endings of the muscles without scarring (Babuccu et al., 2009) and is now regarded as an effective experimental tool for the control of muscle activity similar to that in a clinical environment.

The masticatory muscles power the orofacial system, their structures being closely related to the growth and functions of the teeth, jaw, and joints (Dixon et al., 1997). Although the inhibition of mandibular growth following treatment of BTX to the masticatory muscles has already been addressed (Sakurai et al., 2007; Tsai et al., 2009; Kun-Darbois et al., 2015), the results of these studies may not be comprehensive in that their muscular environments were unnatural, with singular muscular paralysis and two-dimensional analyses. The unilateral singular paralysis of the masticatory muscle may be causing minor structural changes in the mandible and zygoma (Matic et al., 2007) and provoke compensatory work by the medial pterygoid or other muscle (Rafferty et al., 2012). We thus need to consider the simultaneous inhibition of the major masticatory closing muscles, including the masseter, temporal, and medial pterygoid muscles, in order to observe the genuine effect of masticatory muscular inactivity on mandibular growth.

We also paid attention to the possible compensatory reaction of the biological system in terms of bilaterality; the mandibular structure consists of right and left hemi-mandible with articulating joints which are essentially reciprocal, one side of the hemi-mandible influencing and being influenced by the other side (Rafferty et al., 2012). The unilateral masticatory hypofunction may therefore not necessarily produce the same phenotype as that of the bilateral hypofunctional model. In addition, the growth of the nonBTX side in unilateral hypotrophy may not match that of the normal control without any masticatory hypofunction.

Finally, we considered a primate model system to mirror the association between growth and function in human. To infer how human mandibular growth would be impacted by masticatory muscle hypotrophy, we looked for an animal model with craniofacial growth pattern similar to that of human. Investigations to date have documented such similarity in nonhuman hominoids, whose mastication, nasal breathing and orthostatic position affect the mechanical properties of the skeletal system and the mode of facial bone growth (Moss, 1968; Losken et al., 1994). However, they are difficult to work with, which inevitably led us to utilize a primate model system, specifically the cynomolgus monkey, to evaluate masticatory muscle-related growth and function in humans.

The purpose of this study was to investigate the relationship between masticatory muscular function and mandibular skeletal growth. We hypothesized that the mandible would undergo differential growth disturbances in size and shape due to BTX-induced unilateral or bilateral hypotrophy of masticatory muscles. We also introduced synchronous BTX treatments to unilateral or bilateral major masticatory closing muscles to prevent compensatory masticatory muscle function. Three-dimensional (3D) changes in the size and shape of the mandible were obtained by measuring and comparing serial computed tomographic (CT) images.

## Materials and Methods

### Animals and Animal Care

This study was approved by the Animal Experimental Ethics Committee of Southern Medical University, Guangzhou, China (2014-024). All experiments were performed under protocols to meet the requirements of the Association for Assessment and Accreditation of Laboratory Animal Care International and the Experimental Animal Center of Southern Medical University. Male cynomolgus monkeys (*Macaca fascicularis*) were used, preliminary power analysis being performed to get the proper sample size for three groups (G^∗^Power 3.1, Heinrich-Heine-Universität Düsseldorf, Germany; alpha = 0.05, power = 0.95; total *N* = 20 hemi-mandibles). They had complete deciduous dentitions and first permanent molars in occlusion, indicating their juvenile period (McNamara and Graber, 1975).

The animal care was performed by full-time attending veterinarians under the Guide for the Care and Use of Laboratory Animals of the National Institutes of Health. The animals were housed individually in stainless steel cages (85 cm × 92 cm × 100 cm) under a 12 h light-dark cycle at 25–27°C. They were provided with a diet of vitamin enriched biscuits, fruits, and water until they were satisfied. They were also provided with social interactions with neighboring animals and attending veterinarians and given toys and trees for play stimulation. During all experimental periods, they were closely monitored on a regular basis throughout the day by the attending veterinarians as well as the experimental operators to ensure their health and welfare.

For the experimental BTX treatments, the animals were sedated by intramuscular (IM) injection with ketamine HCl (Ketamine, 5–10 mg/Kg, IM) and rompun (Xylazine, 1–2 mg/Kg, IM), with glycopyrrolate (Robinul, 0.004 mg/Kg, IM). Tramadol (50 mg) was given intramuscularly after intervention to alleviate suffering. After 24 weeks of experiments, the animals were sacrificed by a method consistent with the American Veterinary Medical Association Guidelines for the Euthanasia of Animals (IV administration of pentobarbital overdose, >100 mg/Kg) to collect the samples necessary for the histological examination.

### Experimental Design and BTX

The predictor variables were the elapsed time and side of BTX injection; the outcome variables were mandibular structure-related dimensional parameters. Ten monkeys aged 18–24 months (mean age, 22 months), weighing between 2.0 and 3.1 Kg (average 2.44 Kg), were divided into three groups based on the side of BTX injection: group I (control; *n* = 3; mean age 21.0 months), group II (unilateral BTX; *n* = 4; mean age 21.8 months), and group III (bilateral BTX; *n* = 3; mean age 26.7 months) (Supplementary Table S4). The age differences between the groups were not significantly correlated with outcome variables (by Pearson’s product-moment correlation coefficients; details not shown), and the mandibular growth curve by Schneiderman (Schneiderman, 1992) showed a marked decrease in skeletal growth after 4 years old, excluding the possible influence of group age differences.

The masseter, temporal, and medial pterygoid muscles were selected for simultaneous injections of BTX. A total of 20 units/Kg of BTX A (Botulax^®^, Hugel Inc., Chuncheon, Korea, 50 U/ml) was injected into the targeted masticatory muscles. The ratio of the BTX dose to masseter, temporalis, and medial pterygoid muscle was 5:4:3, based on the size and function of the muscle and the therapeutic dose (Bhidayasiri et al., 2006). The muscles on the right (BTX) side were injected for group II (unilateral), and on both sides for group III (bilateral). The masseter and temporalis muscle had two points of injection, while the medial pterygoid had one point. The same amount of normal saline was injected into both sides for group I (control) or into the control side for group II (unilateral). Later, the mandible of group I (control), BTX side of group II (unilateral), control side of group II (unilateral), and group III (bilateral) were independently evaluated for comparison of morphological changes.

Eleven titanium mini-screws (1.2 × 3 mm self-drilling screws; Gssem Co., Korea) were placed at the mandible, maxilla, and cranium as reference markers for 3D superimposition (Bjork, 1955; McNamara and Graber, 1975).

### 3D Morphometric Analysis

#### 3D Imaging

CTs were taken (Aquilion, Toshiba, Japan) at T0 (initial time point just before BTX injection), T1 (3 months after BTX injection), and T2 (6 months after BTX) under sedation. The working condition for CT scan was set to 120 kvp, 150 mA, 0.3 mm of pixel size, and less than 0.5 mm of slice thickness. To maintain the maximum occlusion, the subjects were placed in prone position with the mandible set into a head stabilizer designed for the purpose. CT data were stored in DICOM file format and 3D reconstruction of the mandible and skull and their analyses were performed using software (Mimics and 3-matic, Materialize Co., Leuven, Belgium; Simplant, Materialize Dental Co., Leuven, Belgium).

#### Morphometric Reference Points

Our reference points were selected based on reviews of the morphometric reference points reported so far (Moss and Simon, 1968; Park et al., 2010; Markic et al., 2015), mainly with the aim of facilitating 3D measurements (Supplementary Figures S1A,B). These included the condyle, gonion, and other points for anatomical description as well as nasion, porion, and others for the construction of reference planes. These are detailed in Supplementary Table S1.

#### Reference Planes

The reference planes were defined for linear, angular, area and volume measurements as follows (Supplementary Figures S1C–E and Supplementary Table S2): Frankfort horizontal plane (as the horizontal reference plane), the midsagittal plane (MSP; as the sagittal reference plane), and the coronal plane. The mandibular occlusal plane was set as passing through the mandibular first molars and central incisors (Supplementary Figure S1D), while the mandibular inferior border plane (IBP) was set as passing through the menton-gonion line and running perpendicular to the ramal plane (Supplementary Figure S1E). The mandibular ramal plane runs parallel to the mandibular ramus and passes through the ramus posterior point, sigmoid notch, and ramus anterior point (Supplementary Figure S1E), while the ramus anterior plane is perpendicular to mandibular occlusal plane (Supplementary Figure S1H). Details are described in Supplementary Table S2.

#### 3D Measurements

The dimensional changes of the mandible were evaluated in terms of relative growth increments as well as absolute value changes in lengths, angles, areas and volumes (Supplementary Tables S5–S10). Relative growth rates were calculated by the ratio of measurements at each time period to the size at T0, and then compared with the interval changes from T0 to T1 and T2 (Figure 3–5). We confirmed the possible association between the right and left side of hemi-mandible in group I (control) and group III (bilateral) by paired *t*-test, showing that their differences were not statistically significant (*p* > 0.05; details not shown). We therefore used the hemi-mandibles of groups I and III as independent variables, the average values of right and left side thus being used for statistical analysis.

##### Mandibular unit size

**FIGURE 1:**
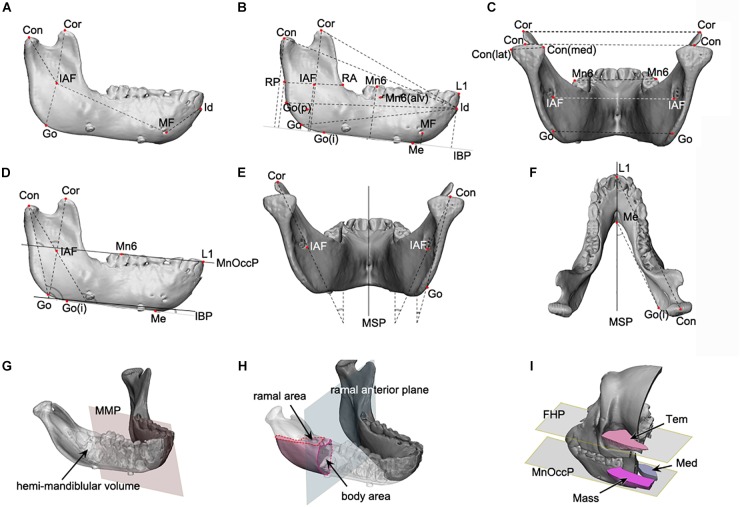
The mandibular linear, angular, area, and volume measurements applied to this study. **(A–C)** The linear measurements; **(D–F)**, the angular measurements; **(G,H)**, the volume and area measurement; **(A)** the mandibular unit measurements, including the condylar and body unit; **(B)** the mandibular length and height measurements; **(C)** the transverse length measurements; **(D)** the angular measurements on the sagittal plane; **(E)** the angular measurements on the coronal plane; **(F)** the angular measurements on the Frankfort horizontal plane; **(G)** the volume measurement of hemi-mandible, produced by the division of mandibular model by mandibular median plane; **(H)** the measurement of cross-sectional area for ramus and body, at the level of mandibular occlusal plane for ramus and ramal anterior plane for body; **(I)** the measurement of cross-sectional area of temporal, medial pterygoid, and masseter muscle, at the mandibular occlusal plane and FHP. Abbreviations are defined in Supplementary Tables S1–S3. Abbreviations for reference points) IAF, inferior alveolar foramen; Con, condyle; Cor, coronoid; Go, gonion; MF, mental foramen; Id, infradentale; Mn6, lower 1st molar; Id, infradentale; Go(p), gonion posterior point; Go(i), gonion inferior point; RA, ramus anterior point; RP, ramus posterior point; Me, menton; Con(med), condylar medial point; Con(lat), condylar lateral point; MF, mental foramen; Li, lower incisor point. Abbreviations for planes: MSP, midsagittal plane; MnOccP, mandibular occlusal plane; IBP, mandibular inferior border plane; FHP, Frankfort horizontal plane; CP, coronal plane; MMP, mandibular median plane; MRP, mandibular ramal plane; RAP, ramus anterior plane. Abbreviations for measurements: IAF-Con, condylar unit; IAF-Cor, coronoid unit; IAF-Go, angular unit; IAF-MF, body unit; Id-MF, symphyseal unit; IBP-Con, condylar height; IBP-Cor, coronoid height; IBP-Go, angular height; IBP-IAF, mandibular foramen height; IBP-Mn6, mandibular molar height; Id-Con, condylar length; Id-Cor, coronoid length; IAF-Go(p), angular length; RA-RP, ramal breadt; Con(med)-Con(lat), condylar head size; Con-Con, condylar width; Cor-Cor, coronoid width; Go-Go, angular width; MF-MF, mental width; Mn6-Mn6, molar width.

The mandible was divided into five units in accordance with the concept of mandibular units or module (Figure 1A; Moss and Young, 1960; Moss, 1968). Each unit was designed to represent developmental characteristics and measured in-line by connecting reference points, as shown in Figure 1A. 3D mandibular unit analysis was performed based on previous reports (Moore, 1973; Delaire, 1990; Park et al., 2010). Lengths were measured for body unit [between the inferior alveolar foramen (IAF) and mental foramen (MF)], condylar unit (condyle point (Con)-IAF), coronoid unit (coronoid point (Cor)-IAF), and others.

##### Mandibular size

The mandible height was measured in terms of distance to each reference point from IBP (Figure 1B). Anterior-posterior (AP) dimension was evaluated as the distance from infradentale (Id) to the reference points (Figure 1B). The mandibular width was measured between the bilateral reference points to obtain condylar width (Con-Con), coronoid width (Cor-Cor) or mandibular first molar width (Mn6-Mn6) (Figure 1C).

##### Mandibular angles

The angles of the mandibular structure were also measured between the reference plane and the ramal axis (Con-Go), mandibular unit (Con-IAF and Cor-IAF), or mandibular border lines (gonion inferior point – Me) (Figure 1D–F).

##### Mandibular volumes and areas

The hemi-mandibular volumes and cross-sectional areas of the ramus (at the level of mandibular occlusal plane) and the body (on the ramus anterior plane) were measured on the constructed 3D models (Figure 1G,H).

##### Masticatory muscular areas

The cross-sectional areas of masseter, medial pterygoid, and temporalis on the reference planes (including mandibular occlusal plane for masseter and medial pterygoid and FHP for temporalis) were measured on the 3D model constructed from CT images using the soft tissue setting (Figure 1I).

#### Assessment of Interval Growth

3D-reconstructed mandibular models for three subsequent time periods were superimposed to verify the chronologic changes using the best-fit algorithm of the software. Three superimposition methods using different registration points were tried to achieve the most accurate picture of dimensional changes (Supplementary Figure S2): the mandibular reference screws, mandibular foramen-mental foramen (IAF-MF), and cranial reference screws. The inter-surface distance between the models was also calculated and color-coded for comparison, based on the superimposition using mandibular reference screws (Figure 6).

#### Histological Analysis

**FIGURE 2:**
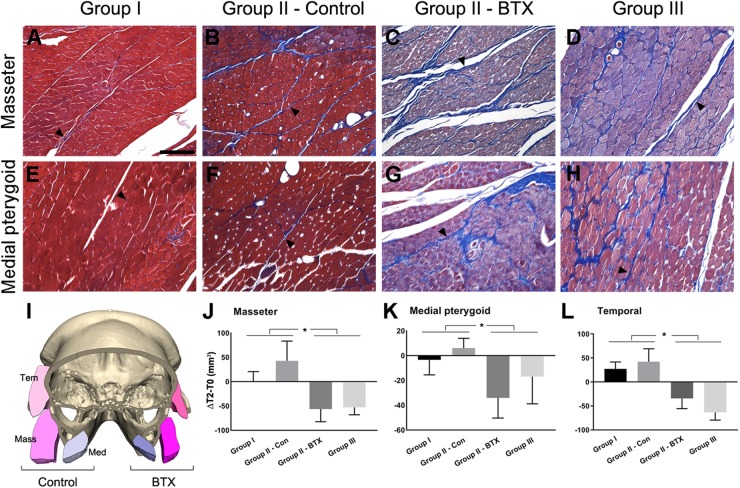
Histological and measurement analysis of masticatory muscles. **(A–H)** Masson’s trichrome staining of masseter **(A–D)** and medial pterygoid muscle **(E–H)** after 6 months of BTX treatments; **(I)**, the comparison of cross-sectional area of muscles in control and the BTX side of group II (unilateral); **(J–L)**, the area measurement of cross-sectional area of muscles and their comparison; **(A,E)** the masseter and medial pterygoid muscles in group I (control), showing reddish myofibers in compact alignment, being encapsulated by thin blue perimysium (arrowhead for perimysium); **(B,F)** the muscles from the control side of group II, showing the similar arrangements of myofibers with more perimysia and dilated vessels; **(C,D,G,H)** masseter and medial pterygoid muscle in group III (bilateral) and on the BTX side of group II (unilateral), which shows the hypotrophic changes with decreased myofiber size and increased perimysia; **(I)** the muscles in the BTX-treated side of group II (unilateral) showed a marked decrease in size as compared with those on the saline-injected control side; **(J–L)** masseter, medial pterygoid, and temporal muscles of group I and control side of group II (unilateral) showed statistically significant greater increments between T0 and T2 than those of group III (bilateral) and the BTX side of group II (unilateral). ^∗^Significant when *p* < 0.05. Scale bar = 50 μm **(A)**.

Following CT evaluation and euthanasia, the head parts of subjects were fixed at room temperature in 10% formalin solution and decalcified in 0.5 M ethylenediaminetetraacetic acid solution at pH 7.4 for 1 year at 4°C. After decalcification, axial sections of masseter and medial pterygoid muscle around the middle point region between ramus anterior and posterior point were obtained. Masson’s trichrome staining (Histoperfect^TM^, Masson’s Trichrome Staining Kit, BBC Biochemical, Stanwood, WA, United States) were used to examine the histological condition of masticatory muscles (Figure 2A–H).

#### Statistical Analysis and Methods Error

Statistical analyses were performed to compare the measurements between the groups. Linear mixed model analysis was used to compare the results in terms of groups, BTX treatments, and time period for bilaterality of mandible. The two-way analysis of variance (ANOVA) test was applied for comparison of measurements between the bilateral structures and verified by *post hoc* analysis with the Bonferroni correction procedure, using Statistical Package for the Social Sciences (SPSS, Version 24, IBM Co.) and Prism (Version 8, Graphpad Co.).

The possible error associated with the methods when assigning reference points on 3D CT was also calculated in each dimension of X, Y, and Z according to Dahlberg’s formula (Dahlberg, 1949). One author (H-JK) digitized each of 5 reference points on 3D CT images 20 times. To evaluate intra-observer variability, a reliability analysis with the determination of the intraclass correlation coefficient (ICC) was calculated with 95% confidence intervals.

## Results

### Body Weight

The mean body weight for all three groups increased from 2.44 Kg (T0) to 2.67 Kg (T2). Specifically, group I (control) gained 0.31 Kg of body weight over 6 months (T2–T0), while weight gain in group II (unilateral) was 0.24 Kg and 0.15 Kg in group III (bilateral). There was no increase in body weight in group III (bilateral) during the first 3 months (T1–T0). While group II (unilateral) and group III (bilateral) gained 0.24 and 0.15 Kg, respectively, Pearson’s correlation analysis revealed no statistically significant difference between body weight and dimensional measurements (*p* > 0.05; details not shown). Subjects in group I (control) had no difficulties feeding, those from group II (unilateral) mainly chewed on the control side, and those from group III (bilateral) temporarily could not eat properly. Group III was thus fed with a full liquid diet through the nasogastric tube for about 2 weeks, followed by normal feeding.

### Histological Analysis

The histological evaluation by Masson’s trichrome staining showed that the masseter and medial pterygoid muscles in group I (control) had red plump myofibers encapsulated by thin or indistinct perimysium (Figure 2A,E; arrowhead for perimysium). The same muscles on BTX side revealed a degenerative hypotrophic change of myofibrils with a decrease in the myofibrillar diameters as well as an increase in the collagen fibers forming perimysium around the myofibers (Figure 2C,D,G,H; arrowheads for perimysium).

### 3D Measurements and Analysis

The mandibular structures were measured and their periodic increments (T2–T0) as well as percentage change with respect to initial values (T2–T0/T0) were calculated to compare time-dependent changes.

#### Mandibular Unit Size

**FIGURE 3:**
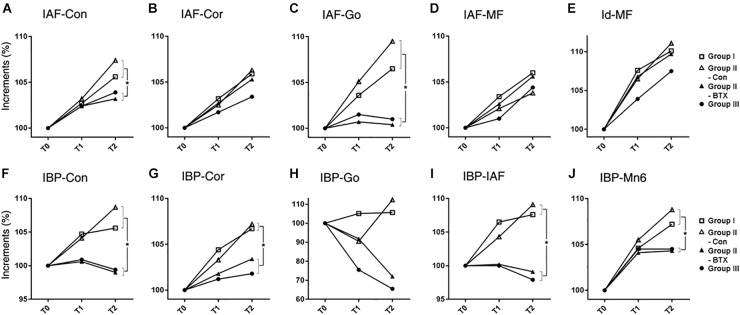
Time-dependent incremental changes in mandibular measurements **(A–E)** mandibular unit size, **(F–J)** mandibular height. Abbreviations: IAF, inferior alveolar foramen; Con, condyle; Cor, coronoid; Go, gonion; MF, mental foramen; Id, infradentale; IBP, mandibular inferior border plane; Mn6, lower 1st molar. Abbreviations: IAF-Con, condylar unit; IAF-Cor, coronoid unit; IAF-Go, angular unit; IAF-MF, body unit; Id-MF, symphyseal unit; IBP-Con, condylar height; IBP-Cor, coronoid height; IBP-Go, angular height; IBP-IAF, mandibular foramen height; IBP-Mn6, mandibular molar height. 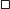 for group I (control), 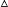 for control side of group II (unilateral), 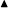 for BTX side of group II (unilateral), 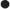 for group III (bilateral). ^∗^Significant when *p* < 0.05, to compare saline- and BTX-treatment by linear mixed model analysis; units in incremental percent (T2–T0/T0).

The increased length at T0–T2 was greatest at the body unit (IAF-MF; 2.0 mm) and least at the angular unit (IAF-Go; 0.8 mm) for group I (control) (Figure 3 and Supplementary Table S5). The relative increase in mandibular unit length for group I (control) was greater than that of other groups, except for the four units on the control side of group II (unilateral). The condylar (IAF-Con) and angular unit (IAF-Go) on the group I (control) and control side of group II (unilateral) showed significantly greater growth increments than on the BTX side of group II (unilateral) and group III (bilateral) (*p* = 0.01 for condylar unit and *p* = 0.04 for angular unit). In contrast, the body (IAF-MF) and coronoid unit (IAF-Cor) showed no significant changes for all groups.

#### Mandibular Size

The periodic increment from T0 to T2 was greatest at the condylar height (IBP-Con) and coronoid height (IBP-Cor) (2.4 mm each) on the control side of group II (unilateral), and least at the angular height (IBP-Go; -0.8 mm) in group III (bilateral) (Supplementary Table S6). The relative growth rate for the coronoid height (IBP-Cor), IAF height (IBP-IAF), and molar height (IBP-Mn6) in group I (control) and the control side of group II (unilateral) was significantly greater than those in group III (bilateral) (*p* < 0.05 for condylar and alveolar height; *p* < 0.01 for coronoid and molar height) (Figure 3).

**FIGURE 4:**
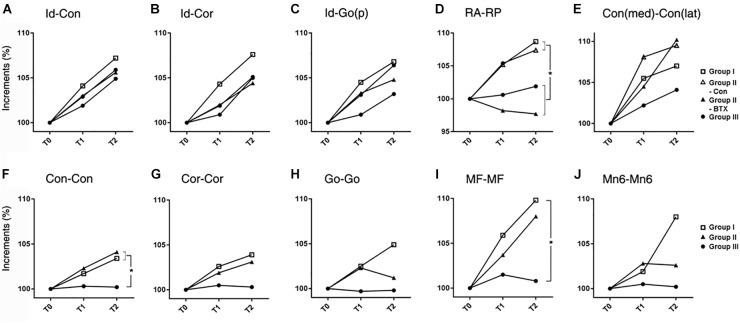
Time-dependent differential changes in mandibular size **(A–E)** mandibular AP length, **(F–J)** mandibular transverse length. Abbreviations: Id, infradentale; Con, condyle; Cor, coronoid; Go(p), gonion posterior point; RA, ramus anterior point; RP, ramus posterior point; Con (med), condylar medial point; Con (lat), condylar lateral point; MF, mental foramen; Mn6, lower 1st molar. Abbreviations: Id-Con, condylar length; Id-Cor, coronoid length; IAF-Go(p), angular length; RA-RP, ramal breadth; Con(med)-Con(lat), condylar head size; Con-Con, condylar width; Cor-Cor, coronoid width; Go-Go, angular width; MF-MF, mental width; Mn6-Mn6, molar width. 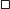 for group I (control), 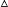 for control side of group II (unilateral), 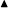 for BTX side of group II (unilateral), 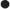 for group III (bilateral). ^∗^Significant when *p* < 0.05, to compare saline- and BTX-treatment by linear mixed model analysis; units in incremental percent (T2-T0/T0).

AP length increments in condylar (Id-Con) and coronoid length (Id-Cor) were not significantly different for all groups, while those of ramal breadth (RA-RP) for group I (control) and control side of group II (unilateral) were significantly greater than those on the group III (bilateral) and BTX side of group II (unilateral) (*p* = 0.01) (Figure 4 and Supplementary Table S7). In addition, the coronoid length (Id-Cor) and condylar head size [Con(med)-Con(lat)] in T2 were significantly greater than that of T0 (*p* < 0.05).

The transverse length at the condylar width (Con-Con) of group I (control) or control side of group II (unilateral) was significantly greater than that of group III (bilateral) and BTX side of group II (unilateral) (*p* < 0.0001 for condylar width and 0.016 for body width) (Figure 4 and Supplementary Table S8). The ramus width (RA-RA) and molar width (Mn6-Mn6) from T0 to T2 were significantly different for all groups.

#### Mandibular Angles

**FIGURE 5:**
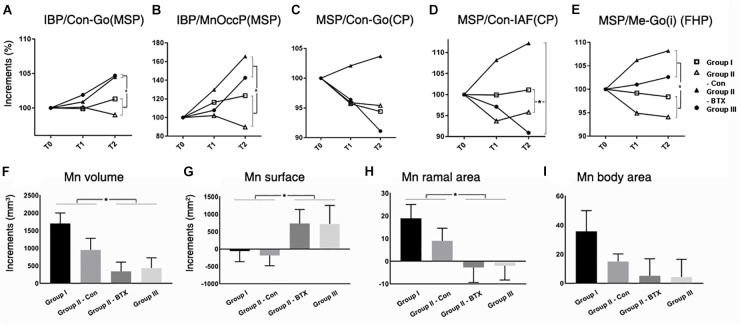
Time-dependent differential changes in mandibular angle and volume measurements for four groups. Abbreviations: IBP, mandibular inferior border plane; Con, condyle; Go, gonion; MSP, midsagittal plane; CP, coronal plane; IAF, inferior alveolar foramen; MnOccP, mandibular occlusal plane; Me, menton; Go(i), gonion inferior point; FHP, Frankfort horizontal plane. Abbreviations: Con-Go, ramal axis; Con-IAF, condylar unit; Me-Go(i), mandibular inferior border line. 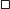 for group I (control), 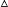 for control side of group II (unilateral), 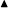 for BTX side of group II (unilateral), 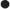 for group III (bilateral). ^∗^Significant when *p* < 0.05, to compare saline- and BTX-treatment by linear mixed model analysis; units in incremental percent (T2–T0/T0) for **(A–E)**, and units in mm^3^ of increments from T0 to T2 (T2–T0) for **(F–I)**.

The angle between the ramal axis (Con-Go) and the IBP on MSP for group III (bilateral) and BTX side of group II (unilateral) was significantly greater than that for group I (control) and control side of group II (unilateral) (*p* = 0.001) (Figure 5 and Supplementary Table S9). The angle between the condylar axis (Con-IAF) and MSP on the coronal plane on the group III (bilateral) and BTX side of group II (unilateral) increased significantly by time periods, moreso than that for group I (control) and control side of group II (unilateral) (*p* = 0.004 for condylar axis). In addition, the angle between MSP and the mandibular inferior border (Me-gonion inferior) on FHP for group III (bilateral) and BTX side of group II (unilateral) was greater than that of group I (control) and control side of group II (unilateral) (*p* < 0.0001) (Figure 1F, 5E and Supplementary Table S9).

#### Mandibular Volumes and Areas

The periodic increment of hemi-mandibular volumes from T0 to T2 was the greatest in group I (1702 mm^3^) and the least on BTX side of group II (949 mm^3^) (Figure 5F and Supplementary Table S10). In addition, the volume increments of BTX treatments in group III (bilateral) and BTX side of group II (unilateral) were significantly smaller than those of saline treatments in group I (control) and control side of group II (unilateral). The difference between the BTX and control sides was also statistically significant for ramal cross-sectional area (Figure 5H and Supplementary Table S10), but insignificant for body cross-sectional area (Figure 5I and Supplementary Table S10).

#### Masticatory Muscular Areas

The cross-sectional areas between T0 and T2 on masseter, medial pterygoid, and temporalis in group I and control side of group II (unilateral) mostly increased, moreso on the control side of group II (Figure 2I–L and Supplementary Table S10). However, those from group III (bilateral) and the BTX side of group II (unilateral) all decreased and were significantly different from saline-treated both sides of group I and the control side of group II (Figure 2J–L and Supplementary Table S10).

### Assessment of Interval Growth

**FIGURE 6:**
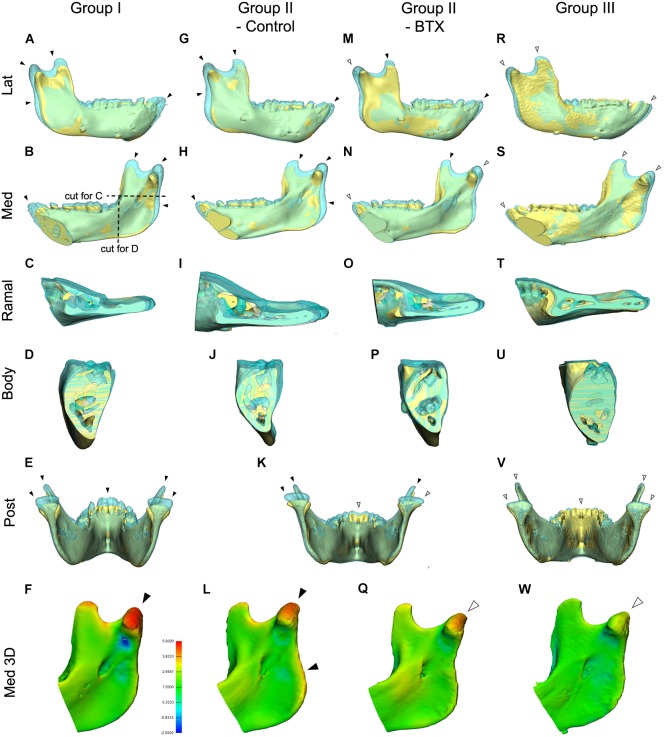
Comparison of mandibular growth of the four groups between stages T0 and T2 based on superimposition at the mandibular screws. **(A–E,G–K,K,M–P,R–V)** The lateral, medial, cross-sectional ramus and body, and posterior sides of the superimposition or section were visualized to compare states T0 and T2. The blue model represents stage T2 mandible, the yellow stage T0. **(F,L,Q,W)** 3D comparison with color-coding to visualize growth-related changes inserted color diagram for figure **(L)** shows the inter-surface distance of the two models. Red indicates bony apposition; blue shows resorption. Arrowheads in **(A–W)**; black for marked growth between T0 and T2; white for less evident changes. Color coding for **(F,L,Q,W)**; in orange and red color for 3.8–5.0 mm; in light and dark blue for -0.9 to -2.0 mm.

Three methods of superimposition in group I (control) yielded different growth measurements (marked with arrowheads; Figure 6). Cranial superimposition by craniomaxillary reference screws (red circles; Supplementary Figure S2) revealed relatively static condylar and posterior ramal regions, with the main direction of growth being at the symphysis and the inferior border (Supplementary Figure S2B). The superimposition at the mandibular foramen and mental foramen showed growth mainly in the condyle, coronoid, posterior ramus, and symphyseal regions, coming midway between the other two methods and matching well with the previously mentioned measurement results (Supplementary Figure S2C). On the other hand, the registration at the mandibular reference screws (marked with red circles) indicated the static inferior border region and the main direction of growth were in the condylar and coronoid regions (Supplementary Figure S2A). We finally chose the fixed screw-based superimposition method to evaluate growth changes.

Figure 6 shows the superimposed mandibular models of stages T0 and T2 based on screw superimposition for comparison of dimensional changes among the four groups. The lateral and medial views for group I (control) and the control side of group II (unilateral) showed greater dimensions at T2 for the condylar, coronoid, posterior ramus, and symphyseal regions (shown in blue and indicated by black arrowheads), showing marked growth in the superior, posterior, and anterior growth directions (Figure 6A–L). They also showed less evident changes in the inferior border, dentoalveolus, and lateral ramus regions, indicating less growth in the inferior, lateral, and dental areas (shown in blue and indicated by white arrowheads). In addition, the control side of group II (unilateral) had more growth in the posterior ramus and angular regions than did group I (control) (Figure 6A–E,G–K). The same views of the BTX side of group II (unilateral) and group III (bilateral) presented a minimal inter-surface distance in all corresponding regions, including the inferior border, compared to that in group I (control) (Figure 6K,M–Q for group II (BTX); Figure 6R–W for group III (bilateral). In addition, group III (bilateral) had the least eruption at the anterior teeth without accompanying open bite. These changes were also confirmed by color-coded distance measurements as seen in Figure 6F,LQ,W.

The posterior and cross-sectional views in group I (control) showed greater increments and lateral-posterior direction of growth in the condylar, coronoid, and posterior ramus and maintaining angular interrelationships and sectional area of body being insignificant changes (Figure 6B–E). This condylar lateral expansive growth with increased width was not found in group II (unilateral) or group III (bilateral) (Figure 6I–K,O,P). Moreover, the BTX side of group II (unilateral) showed a lateral tilt of condyle and posterior ramus with a lateral elongated condylar head (Figure 6K).

### Methods Error

The methods error when assigning reference points was 0.05 mm on average, the ICC being 0.972 (by Cronbach’s alpha).

## Discussion

This study aimed to evaluate the association between masticatory muscle function and mandibular growth in juvenile primates. The function of major masticatory closing muscles was simultaneously interrupted by BTX treatment in the unilateral or bilateral muscles of growing cynomolgus monkeys. Mandibular growth evaluated with 3D CT data at three time points over 6 months showed dimensional and angular changes in skeletal structural growth, mainly in the ramal area in the vertical dimension.

The mandibular and craniofacial growths of nonhuman primates, especially genus Macaca, have been studied in depth (Siegel et al., 1985). The juvenile monkey at age 18–24 months has a deciduous dentition with the first permanent molars in full occlusion (McNamara and Graber, 1975). The growth velocity of the Macaca mandible peaks at 3 years of age on average, and drops after 4 years old (McNamara and Graber, 1975). The ages of our models ranged 21–27 months and the experiments continued for 6 months, covering a period of rapid juvenile growth without significant differences in growth speed among groups.

The mandible is a U-shaped structure mainly consisting of the ramal and body parts. The ramal structure, particularly in mammals, consists of one angular region and two processes, forming a Y-shape. The plump masticatory muscles, i.e., masseter, temporal and medial pterygoid muscles, envelop the ramus and provide stability and mobility as well as the main blood supply to the ramus (Cohen, 1960; Saka et al., 2002).

The normal growth and maintenance of the mandibular skeletal structure is influenced by various genetic as well as epigenetic factors such as masticatory muscular force and function, bite force, chewing pattern, and diet (Dechow and Carlson, 1990). The decreased loading with soft diet influences the internal structure, bone mass, cortical thickness, and morphology of mandible (Kiliaridis, 1995). It also results in a posteriorly rotated mandible, reduced ramal size, more posteriorly directed growth of the condyle, and a shorter vertically angular process (Bouvier and Hylander, 1984; Kiliaridis et al., 1985; Yamada and Kimmel, 1991), or limited vertical displacement (Kono et al., 2017), similar to the results of BTX treatments in our group III (bilateral).

BTX paralyzes muscles by blocking acetylcholine release at the neuro-muscular junction, their recovery being accomplished by motor axon sprouts (Angaut-Petit et al., 1990). The time elapsed for the recovery varies by animal (Pickett, 2012), but the partial or complete recovery of masticatory function comes around 3 months in human, mouse, and rabbit (Poliachik et al., 2010; Park et al., 2013). However, some studies report a delay in muscular and skeletal recovery of 3 or 6 months after BTX to monkey masseter (Capra et al., 1991), mouse limb muscle (Grimston et al., 2007) and human calf muscles (Polak et al., 2002). In addition, our evaluations of muscles morphologically, histologically, radiographically, and functionally at the end of experiment clearly indicated incomplete recovery of masticatory muscles after 6 months. Furthermore, incremental changes in body weight and the major measurement variables showed no clear evidence suggesting prominent muscular recovery after 3 months of BTX treatment.

The most common physiological test to determine jaw muscle activity is electromyography, but muscle measurement (length, thickness, cross-sectional area, and volume) constitutes an alternative method using ultrasonography, computed tomography, or magnetic resonance imaging. The cross-sectional area of jaw muscle and bite force magnitude are closely related (van Spronsen et al., 1989). In order to determine the state of the tested masticatory muscles, we measured the cross-sectional area of three masticatory muscles at the level of the mandibular occlusal plane and FHP on CT images of T0 and T2. The masticatory muscles all decreased in cross-sectional area with statistical significance by BTX treatment, augmenting the histological evaluation of muscle hypotrophy.

The functional relationship between muscle and bone can be observed throughout mandibular growth, development, and aging (Hamrick, 2010). It has long been advocated that function and form are closely related (Sugiyama et al., 2002), and that functional stress patterns bone morphology (Wolff, 1892). Moss described the skeletal unit as being biomechanically supported and/or protected by its related functional matrix (Moss, 1968), and Frost suggested that bone strength and mass are controlled by mechanically-loaded strain (Frost, 1998). A significant association was proved between mandibular shape and muscle cross-sectional areas, such as the association between the larger muscular cross-sectional area and more trapezoidal ramus and rectangular body with more massive coronoid (Sella-Tunis et al., 2018). Our results showing reduced muscular and mandibular skeletal cross-sectional areas in BTX treatment groups also support this functional relationship between muscle and bone.

Our measurement results showed that the ramus, especially in the vertical dimension, was the main region for structural changes following BTX treatment, with remarkable structural differences between the BTX- and saline-applied angular, condylar, coronoid, and posterior ramus regions after 6 months. This was first evidenced by measurements of the angular region, including the angular unit (IAF-Go) and mandibular foramen height (IBP-IAF). For example, the mandibular angular unit lengths (IAF-Go) of group III (bilateral) and the BTX side of group II (unilateral) showed stagnant growth (0.1 mm, 0.8% each), while the lengths of group I (control) and the control side of group II (unilateral) increased (0.8 mm, 6.5%; 1.2 mm, 9.5%) (Supplementary Table S5). These regional changes were, similarly, observed in the superimpositions and color-coded measurements of 3D models for group II (unilateral) and group III (bilateral) (Figure 6). This result was consistent with reports of other studies that the ramus height decreased after BTX injection into the masticatory muscles of rats and rabbits (Kwon et al., 2007; Tsai et al., 2009).

The mandibular angle is the region of masseter and medial pterygoid muscle attachment. It is known as a secondary and mechanically obligatory region that can respond to the functional demands of these muscles (Moss and Simon, 1968). Normal remodeling in the angular region in primates has already been reported (McNamara and Graber, 1975), despite a gradual decrease with age (Bravo et al., 1989). We assumed that BTX decreased muscle function, thereby inhibiting growth in the mandibular angle area, while the compensatory action of the control side of group II (unilateral) accelerated growth in the angular region.

The most prominent incremental change in normal growth as well as in BTX-induced hypoplastic growth was in the condylar and coronoid regions, and it was again in the vertical dimension. This was evidenced in the results for the condylar unit (IAF-Con), condylar height (IBP-Con), and coronoid height (IBP-Cor). For instance, the condylar height (IBP-Con) of group III (bilateral) and the BTX side of group II (unilateral) showed stagnation or slight decrease (-0.2 mm, -0.7 %; -0.3 mm, -1.1%, respectively), while group I (control) and the control side of group II (unilateral) showed a distinct increase (1.6 mm, 5.6%; 2.4 mm, 8.6 %) (Supplementary Table S6). The condylar head had more distinct BTX-related inhibitory undergrowth (as shown in yellow-orange color of Figure 6Q,W) and compensatory overgrowth (shown in red, Figure 6L) than did the coronoid or other regions. This suggests a stronger association between masticatory function and growth of the condyle than of the coronoid or other regions, though the coronoid has the major masticatory muscle attachment. This may be related to reports that the chondrogenic growth potential of the condylar head, especially the articular portion, under indirect loading is greater than that of the coronoid region (Miyazaki et al., 2016).

One more interesting result was the remarkable growth on the control side of group II (unilateral), possibly induced by compensatory unilateral mastication on the contralateral side. Many previous studies applied BTX unilaterally, using the other side as the control (Kwon et al., 2007; Tsai et al., 2009). However, our results clearly showed the presence of compensatory function and point out the need to differentiate the effects of muscular hypotrophy and compensatory outcomes. In addition, unbalanced growth on the nonBTX side may accentuate asymmetrical growth in that it accompanies decreased growth on the BTX side. A possible etiopathogenic association with human asymmetric growth needs further clarification.

The BTX-induced vertical dimensional changes in the anterior part of the mandible were less evident than those in the ramal part. The indices for anterior vertical dimension, such as the mental foramen height (IBP-MF), incisor height (IBP-Id) and molar height (IBP-Mn6), demonstrated similar small increases within the range of 0.2-1.2 mm (4.3–15.3%) in group III (bilateral) and the BTX side of group II (unilateral), as compared with a range of 0.2-1.6 mm (5.4–8.5%) in group I (control) and the control side of group II (unilateral). The cross-sectional areas of the body region were not significantly different for all groups, as compared with ramus region results. These indifferent growth increments in the anterior mandibular part might indicate less involvement of masticatory muscles in this region than in the ramus region. It is not clear whether they are related to the limited contribution of masticatory muscle or functional matrix that mainly envelop the ramal region (Hohl, 1983).

The normal growth ranges in the AP dimension were relatively greater than those in the vertical dimension. While the condylar height (IBP-Con) and the coronoid height (IBP-Cor) in group I (control) showed a 1.6 and 1.3 mm increase over 6 months, the condylar AP length (Id-Con) and angular AP length [Id-Go (p)] in group I increased by 4.5 and 4.4 mm. Our AP growth increments were similar to those of a previous study (Schneiderman, 1992), which reported 3.77 mm at the same point per 6 months between 1.5 and 3.5 years of age. However, the AP dimension growth changes in the BTX-treatment groups were not so different from those in the control group. The lengths of the condylar AP (Id-Con), angular AP (Id-gonion posterior point), coronoid AP (Id-Cor) and the symphyseal unit (Id-MF) on the BTX-treated side showed growth increments similar to those of the control side. These results indicate a smaller contribution of masticatory muscle to AP growth (Kiliaridis, 1995).

One more notable finding regarding AP length again concerns the ramus. The ramal breadths (RA-RP) of group I (control) and the control side of group II (unilateral) increased (1.6 mm, 8.6%; 1.2 mm, 7.4%), but decreased 0.4 mm (2.3%) on the BTX side of group II (unilateral) and increased only 0.4 mm (1.8%) for group III (bilateral) (Supplementary Table S7). These changes are also evident in the superimposed 3D models and their cross-sectional views, which show a lack of apposition on the posterior border of ramus on the BTX side of group II (unilateral) and group III (bilateral). Bone resorption, which has been well documented on the anterior border of ramus and apposition on the posterior border, is related to the progressive process of mandibular relocation and tooth eruption (Krarup et al., 2005). These changes suggest the contribution of masticatory muscle to the control of ramal breadth either directly or indirectly.

The angulations of the ramus [IBP/Con-Go (MSP)] and occlusal plane [IBP/MnOccP (MSP)] on the sagittal plane for group I (control) and control side of group II (unilateral) were markedly constant during the whole experimental period (Figure 5, 6 and Supplementary Table S9), the condyle showing limited changes within the ranges of 0.8 and 1.9 degrees. However, angulations on the BTX side of group II (unilateral) and group III (bilateral) were significantly different in that they increased 3.1–5.2 degrees over the same period. They indicate that hypotrophy of the masticatory muscles induced a more posterior tilt of the ramus/condyle and a greater clockwise rotation of the occlusal plane. They also suggest the effect of masticatory muscle loading on the sagittal relationship between the ramus and body, as well as between the body and dentoalveolus. Although underlying pathogeneses remain unclear, muscular function may be involved; further evaluation is planned in this regard.

Angular measurements on the coronal plane were similar to those on the sagittal plane in that the angulation of group I subjects were relatively unchanged over the experiment period (Figure 6 and Supplementary Table S9). This meant a constant lateral and superior axis of growth along the condylar and ramal axes on the coronal plane. The condylar growth direction is well known to be V-shaped (Enlow and Harris, 1964). The condyle of group I subjects grew in the posterior-superior direction with an almost 1:1 ratio of horizontal and vertical displacement, as previously reported (McNamara and Graber, 1975; Nanda et al., 1987).

However, the altered masticatory muscle function also accompanied angular changes of the mandible on the coronal plane, as seen in the condylar unit angle (MSP/Con-IAF (CP)); these increased on the BTX side of group II (unilateral) and group III with statistical significance. These results suggest the coincidental development of mandibular asymmetry and lateral tilting due to unilateral muscular function. The phenotype of human asymmetry closely matches that of group II (unilateral) in presenting a ramus canted toward the short ramal side. Greater lateral angulation on the BTX side may be amplified by the lateral path of closure due to BTX-induced unilateral mastication (Lepley et al., 2010).

## Conclusion

The impact of masticatory muscle function on mandibular growth was investigated. Following BTX-induced synchronous hypotrophy of the masticatory muscles of juvenile nonhuman primates, the ramus growth decreased mainly in the vertical dimension with compensatory growth on the control side of unilateral treatment. The BTX side of unilateral treatment showed increased posterior tilt (increased sagittal angles) and lateral tilt (increased coronal angles) of the ramus. However, AP growth following unilateral or bilateral BTX-treatment was similar to that of group I (control), except at the ramal breadth.

In conclusion, BTX-induced masticatory muscle hypotrophy resulted in a decrease in the size of the mandible and also a change in its form, particularly in the vertical dimension and in the posterior-lateral angulation of the ramus. It also accompanied compensatory growth of the nonBTX side ramus that might accentuate asymmetrical growth of the hypofunctional mandible.

## Ethics Statement

This study was approved by the Animal Experimental Ethics Committee of Southern Medical University, Guangzhou, China (2014-024). All experiments were performed under protocols to meet the requirements of the Association for Assessment and Accreditation of Laboratory Animal Care International and the Experimental Animal Center of Southern Medical University.

## Author Contributions

H-JK, ZP, and S-HL designed the study. H-JK, J-WM, H-JT, JH, and ZP did the experiments and data acquisition. J-WM, H-JT, S-HK, STK, H-JK, and S-HL performed analysis and interpretation of data. H-JK and S-HL wrote the draft of the manuscript. S-HK and STK contributed to revise and all authors approve the manuscript to be published and agreed on all aspects of the work.

## Conflict of Interest Statement

The authors declare that the research was conducted in the absence of any commercial or financial relationships that could be construed as a potential conflict of interest.

## Supplementary Material

FIGURE S1 The reference points and planes for the measurements. **(A,B)** the mandibular landmark points designed for measurements, revealed on the lateral **(A)** and posterior **(B)** views; **(C–E)** the cranial and mandibular reference planes used in this study. Abbreviations are defined in Supplementary Tables S1, S2. Abbreviations: IAF, inferior alveolar foramen; Con, condyle; Cor, coronoid; Go, gonion; MF, mental foramen; Id, infradentale; Mn6, lower 1st molar; Id, infradentale; Go(p), gonion posterior point; Go(i), gonion inferior point; RA, ramus anterior point; RP, ramus posterior point; Me, menton; Con(med), condylar medial point; Con(lat), condylar lateral point; MF, mental foramen; Li, lower incisor point. Abbreviations: MSP, midsagittal plane; MnOccP, mandibular occlusal plane; IBP, mandibular inferior border plane; FHP, Frankfort horizontal plane; CP, coronal plane; MMP, mandibular median plane; MRP, mandibular ramal plane.

FIGURE S2 Comparison of three superimposition methods showing different growth patterns for group I (control) model. **(A)** the mandibular registration at the screws on the inferior border showing the superior growth direction in the condylar and coronoid regions; **(B)** the cranial screw-based cranial superimposition showing the main growth direction at the symphysis and on the inferior border; **(C)** superimposition at the mandibular and mental foramen showing growth in the condyle, coronoid, posterior ramus, and symphyseal regions. Red circles indicate the position of reference screws and points of superimpositions.

TABLE S1 The landmark points and their descriptions used in this study.

TABLE S2 The reference planes and their descriptions used in this study.

TABLE S3 Mandibular measurements for height, length, width, angles, areas, and volume.

TABLE S4 Age and change of body weight by group.

TABLE S5 The measurements of mandibular unit.

TABLE S6 Mandibular height measurements.

TABLE S7 Mandibular length measurements.

TABLE S8 Mandibular width measurements.

TABLE S9 Mandibular angular measurements.

TABLE S10 Mandibular volume and area measurements.
