# Supplementary figures and images for: Mandibular Vertical Growth Deficiency After Botulinum-Induced Hypotrophy of Masticatory Closing Muscles in Juvenile Nonhuman Primates
Source: Front Physiol. 2019 Apr 26;10:496. doi: 10.3389/fphys.2019.00496 (PMC6497797; doi:10.3389/fphys.2019.00496)

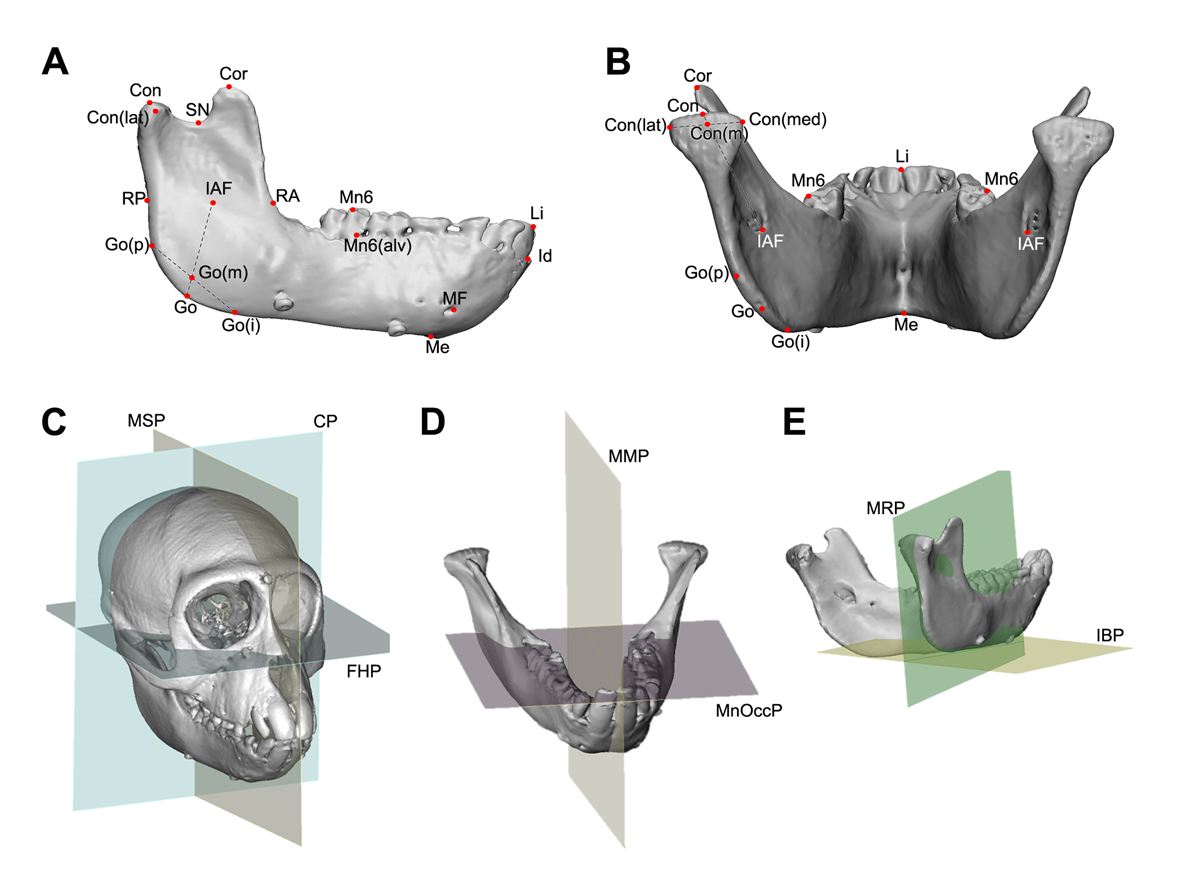

Supplement: FIGURE S1 — The reference points and planes for the measurements. (A,B) the mandibular landmark points designed for measurements, revealed on the lateral (A) and posterior (B) views; (C–E) the cranial and mandibular reference planes used in this study. Abbreviations are defined in Supplementary Tables S1, S2. Abbreviations: IAF, inferior alveolar foramen; Con, condyle; Cor, coronoid; Go, gonion; MF, mental foramen; Id, infradentale; Mn6, lower 1st molar; Id, infradentale; Go(p), gonion posterior point; Go(i), gonion inferior point; RA, ramus anterior point; RP, ramus posterior point; Me, menton; Con(med), condylar medial point; Con(lat), condylar lateral point; MF, mental foramen; Li, lower incisor point. Abbreviations: MSP, midsagittal plane; MnOccP, mandibular occlusal plane; IBP, mandibular inferior border plane; FHP, Frankfort horizontal plane; CP, coronal plane; MMP, mandibular median plane; MRP, mandibular ramal plane. [file Image_1.TIF]

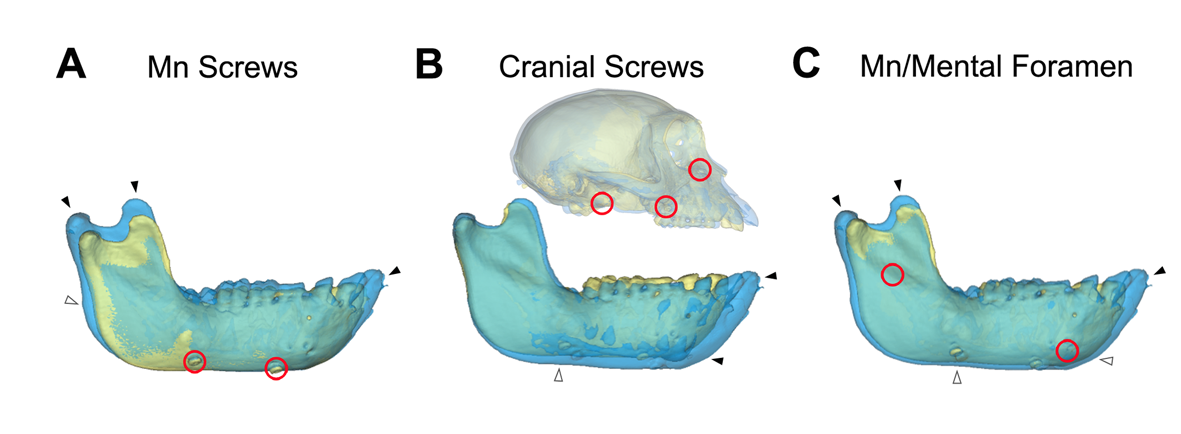

Supplement: FIGURE S2 — Comparison of three superimposition methods showing different growth patterns for group I (control) model. (A) the mandibular registration at the screws on the inferior border showing the superior growth direction in the condylar and coronoid regions; (B) the cranial screw-based cranial superimposition showing the main growth direction at the symphysis and on the inferior border; (C) superimposition at the mandibular and mental foramen showing growth in the condyle, coronoid, posterior ramus, and symphyseal regions. Red circles indicate the position of reference screws and points of superimpositions. [file Image_2.TIF]
