# Supplementary material for: Mandibular Vertical Growth Deficiency After Botulinum-Induced Hypotrophy of Masticatory Closing Muscles in Juvenile Nonhuman Primates
Source: Front Physiol. 2019 Apr 26;10:496. doi: 10.3389/fphys.2019.00496 (PMC6497797; doi:10.3389/fphys.2019.00496)
Supplement: TABLE S1 — The landmark points and their descriptions used in this study. [file Table_1.docx]

Table S1. The landmark points and their descriptions used in this study.

| Point | Name | Description |
| --- | --- | --- |
| Na | Nasion | Junction of frontal and nasal bones on the midline |
| Or | Orbitale | The most inferior point of orbital rim |
| Po | Porion | The most superior point of external auditory meatus |
| Ba | Basion | Midpoint on anterior margin of foramen magnum of  occipital bone |
|  |  |  |
| Bregma | Bregma | Intersection of coronal and sagittal suture of cranial vault |
| Con(lat) | Condylar lateral pole | The most lateral point of condylar head |
| Con(med) | Condylar medial pole | The most medial point of condylar head |
| Con | Condyle | Intersection of condylar line and condylar head |
| Con(m) | Condylar midpoint | Midpoint of condylar medial and lateral pole |
| Cor | Coronoid | The most superior point of coronoid process |
| Go(m) | Gonion midpoint | Midpoint of gonion posterior and inferior point |
| Go(i) | Gonion inferior | The most inferior point of mandibular gonion |
| Go(p) | Gonion posterior | The most posterior point of mandibular gonion |
| Go | Gonion | Intersection of gonial line and mandibular angle |
| Me | Menton | The most inferior point on mandibular symphysis |
| IAF | Inferior alveolar  foramen | The most inferior-anterior point of inferior alveolar foramen  opening |
| MF | Mental foramen | The most anterior point of mental foramen |
| Li | Lower incisor | Contact point of right and left lower central incisor |
| Id | Infradentale | Middle point at superior tip on septum between  mandibular central incisors |
| Mn6(alv) | Alveolar  bone point of lower first  molar | Mesiobuccal alveolar crest point of lower first molar |
| Mn6 | Lower first molar | Mesiobuccal cusp tip of lower first molar |
| RA | Ramus anterior point | Anterior point of ramus (as contact point of anterior  oblique ridge and mandibular occlusal plane) |
| RP | Ramus posterior point | The most posterior and concave area of posterior ramus |
| SN | Sigmoid notch | The most concave area of sigmoid notch |

Details can be seen in association with Figure S1A and B.
