# Supplementary material for: Mandibular Vertical Growth Deficiency After Botulinum-Induced Hypotrophy of Masticatory Closing Muscles in Juvenile Nonhuman Primates
Source: Front Physiol. 2019 Apr 26;10:496. doi: 10.3389/fphys.2019.00496 (PMC6497797; doi:10.3389/fphys.2019.00496)
Supplement: TABLE S2 — The reference planes and their descriptions used in this study. [file Table_2.docx]

Table S2. The reference planes and their descriptions used in this study.

| Plane | Name | Description |
| --- | --- | --- |
| MSP | Midsagittal plane | Plane passing through nasion, bregma, and basion |
| FHP | Frankfort horizontal plane | Plane passing through both sides of porion and midpoint of orbitale |
| CP | Coronal plane | Plane perpendicular to MSP and FHP, passing through basion |
| MRP | Mandibular ramal plane | Plane passing through sigmoid notch, ramus anterior and posterior point |
| IBP | Mandibular inferior border plane | Plane perpendicular to MRP and passing through gonion inferior and menton |
| MMP | Mandibular median plane | Plane perpendicular to FHP, passing through midpoint of Inferior alveolar foramen and midpoint of mental foramen |
| MnOccP | Mandibular Occlusal plane | Plane passing through both sides of lower first molar and infradentale |
| RAP | Ramus Anterior Plane | Plane passing through RA & perpendicular to MnOccP |

Details can be seen in association with Figure S1C-E.
