# Supplementary material for: Mandibular Vertical Growth Deficiency After Botulinum-Induced Hypotrophy of Masticatory Closing Muscles in Juvenile Nonhuman Primates
Source: Front Physiol. 2019 Apr 26;10:496. doi: 10.3389/fphys.2019.00496 (PMC6497797; doi:10.3389/fphys.2019.00496)
Supplement: TABLE S3 — Mandibular measurements for height, length, width, angles, areas, and volume. [file Table_3.docx]

Table S3. Mandibular measurements for height, length, width, angles, areas, and volumes.

| Mandibular unit | Height | Length | Width | Angle | Volume/Area |
| --- | --- | --- | --- | --- | --- |
| IAF-Con | IBP-Con | Id-Con | Con-Con | IBP/Con-Go (MSP) | Hemi-mandibular volume |
| IAF-Cor | IBP-Cor | Id-Cor | Cor-Cor | IBP/Con-IAF (MSP) | Ramal cross-sectional area |
| IAF-Go | IBP-Go | Id-Go | Go-Go | IBP/Cor-IAF (MSP) | Body cross-section area |
| IAF-MF | IBP-IAF | Id-Go(p) | IAF-IAF | IBP/MnOccP (MSP) | Temporal cross-sectional area |
| Id-MF | IBP-Id | Id-RA | MF-MF | Con-Go/MnOccP (MSP) | Masseter cross-sectional area |
|  | IBP-Mn6 | RA-RP | Mn6-Mn6 | Con-IAF/Cor-IAF (MSP) | Medial pterygoid cross-sectional area |
|  | IBP-MF |  | RA-RA | MSP/Con-Go (CP) |  |
|  |  |  | Con(med)-Con(lat) | MSP/Con-IAF (CP) |  |
|  |  |  |  | MSP/Cor-IAF (CP) |  |
|  |  |  |  | MSP/Me-Go(i) (FHP) |  |
|  |  |  |  | MSP/L1-Con (FHP) |  |

Details can be seen in association with Figure 1.
