# Supplementary material for: Mandibular Vertical Growth Deficiency After Botulinum-Induced Hypotrophy of Masticatory Closing Muscles in Juvenile Nonhuman Primates
Source: Front Physiol. 2019 Apr 26;10:496. doi: 10.3389/fphys.2019.00496 (PMC6497797; doi:10.3389/fphys.2019.00496)
Supplement: TABLE S4 — Age and change of body weight by group. [file Table_4.docx]

Table S4. Age and change of body weight by group.

|  | Age | Body weight | | | |
| --- | --- | --- | --- | --- | --- |
|  | T0 | T0 | T1 | T2 | ΔT0-T2 |
| Group I (control) | 21.0±2.65 | 2.42±0.19 | 2.63±0.15 | 2.73±0.23 | 0.31±0.15 |
| Group II (unilateral) | 21.8±3.86 | 2.16±0.20 | 2.35±0.25 | 2.40±0.24 | 0.24±0.05 |
| Group III (bilateral) | 26.7±2.31 | 2.82±0.33 | 2.83±0.35 | 2.97±0.26 | 0.15±0.13 |
| Average | 23.2±3.77 | 2.44±0.36 | 2.60±0.31 | 2.67±0.31 | 0.23±0.16 |

Units in months for age and in kilograms for body weight.
