# Supplementary material for: Mandibular Vertical Growth Deficiency After Botulinum-Induced Hypotrophy of Masticatory Closing Muscles in Juvenile Nonhuman Primates
Source: Front Physiol. 2019 Apr 26;10:496. doi: 10.3389/fphys.2019.00496 (PMC6497797; doi:10.3389/fphys.2019.00496)
Supplement: TABLE S5 — The measurements of mandibular unit. [file Table_5.docx]

Table S5. The measurements of mandibular unit.

|  | Group I (control) | | | Group II (unilateral) | | | Group II (unilateral) | | | Group III (bilateral) | | | *p** | *p†* | *p‡* |
| --- | --- | --- | --- | --- | --- | --- | --- | --- | --- | --- | --- | --- | --- | --- | --- |
|  |  |  |  | - control side | | | - BTX side | | |  |  |  |  |  |  |
|  | T0 | T1 | T2 | T0 | T1 | T2 | T0 | T1 | T2 | T0 | T1 | T2 |  |  |  |
| IAF-Con | 19.7±9.7 | 20.3±0.3 | 20.8±0.8 | 19.1±9.1 | 19.7±9.7 | 20.5±0.5 | 19.3±9.3 | 19.8±9.8 | 19.9±9.9 | 19.9±9.9 | 20.4±0.4 | 20.7±0.7 | 0.52 | 0.01 | 0.48 |
| IAF-Cor | 21.2±1.0 | 21.9±1.9 | 22.5±2.5 | 20±0.5 | 20.6±0.6 | 21.3±1.3 | 20.1±0.1 | 20.6±0.6 | 21.1±1.1 | 21.4±1.0 | 21.8±1.8 | 22.1±2.1 | 0.76 | 0.62 | 0.86 |
| IAF-Go | 12.3±2.3 | 12.7±2.7 | 13.1±3.1 | 12.5±2.5 | 13.2±3.2 | 13.7±3.7 | 12.4±2.4 | 12.5±2.0 | 12.5±2.5 | 13±3.5 | 13.2±3.2 | 13.1±3.1 | 0.77 | 0.04 | 0.16 |
| IAF-MF | 34.3±4.3 | 35.4±5.4 | 36.3±6.3 | 34.4±4.4 | 35.1±5.1 | 35.7±5.7 | 33.2±3.2 | 34.1±4.1 | 35.1±5.1 | 35.8±5.8 | 36.2±6.2 | 37.4±7.4 | 0.39 | 0.30 | 0.53 |
| Id-MF | 18.1±8.1 | 19.4±9.4 | 19.9±9.9 | 15.9±5.0 | 17±7.9 | 17.7±7.7 | 16.5±6.5 | 17.6±7.6 | 18.1±8.1 | 19.5±9.0 | 20.3±0.0 | 21±1.3 | 0.96 | 0.71 | 0.01 |

Units in mm; T0 for initial stage; T1 for second stage three months after initiation of experiment; T2 for final stage six months after initiation of experiment.

significant when p < 0.05 by linear mixed model analysis.

*p** for comparison of groups between group I, II and III; *p†* for comparison of saline- and BTX-treated side; *p‡* for comparison of time-related changes between T0, T1 and T2

Details can be seen in association with Figure 1A and 3 and Table S3.
