# Supplementary material for: Mandibular Vertical Growth Deficiency After Botulinum-Induced Hypotrophy of Masticatory Closing Muscles in Juvenile Nonhuman Primates
Source: Front Physiol. 2019 Apr 26;10:496. doi: 10.3389/fphys.2019.00496 (PMC6497797; doi:10.3389/fphys.2019.00496)
Supplement: TABLE S6 — Mandibular height measurements. [file Table_6.docx]

Table S6. Mandibular height measurements.

|  | Group I (control) | | | Group II (unilateral) | | | Group II (unilateral) | | | Group III (bilateral) | | | *p** | *p†* | *p‡* |  |  |
| --- | --- | --- | --- | --- | --- | --- | --- | --- | --- | --- | --- | --- | --- | --- | --- | --- | --- |
|  |  |  |  | - control side | | | - BTX side | | |  |  |  |  |  |  |  |  |
|  | T0 | T1 | T2 | T0 | T1 | T2 | T0 | T1 | T2 | T0 | T1 | T2 |  |  |  |  |  |
| IBP-Con | 28.5±0.8 | 29.9±1.1 | 30.1±1.5 | 27.9±1.0 | 29±1.3 | 30.3±1.4 | 27.7±1.0 | 27.9±0.7 | 27.4±0.7 | 27.7±0.7 | 28±0.8 | 27.5±1.5 | 0.31 | 0.00 | 0.01 |  |  |
| IBP-Cor | 34.3±0.8 | 35.8±0.8 | 36.6±0.6 | 32.9±1.8 | 34±2.1 | 35.3±2.3 | 32.5±1.6 | 33.1±1.9 | 33.6±1.8 | 34.4±0.8 | 34.8±0.8 | 35±1.0 | 0.54 | 0.01 | 0.21 |  |  |
| IBP-Go | 2.0±0.4 | 2.1±0.5 | 2.2±0.5 | 1.7±0.1 | 1.5±0.2 | 1.9±0.5 | 1.6±0.1 | 1.5±0.3 | 1.2±0.3 | 2.2±0.3 | 1.6±0.5 | 1.4±0.6 | 0.77 | 0.11 | 0.36 |  |  |
| IBP-IAF | 13.5±0.7 | 14.4±0.6 | 14.5±0.9 | 13.2±0.8 | 13.8±0.5 | 14.4±0.5 | 13.1±1.0 | 13.1±0.8 | 13±1.0 | 13.9±1.2 | 13.9±1.1 | 13.6±1.6 | 0.83 | 0.00 | 0.07 |  |  |
| IBP-Id | 13.4±0.4 | 14.3±0.7 | 14.5±1.0 | 11.8±1.4 | 12.5±1.3 | 12.7±1.6 | 11.5±1.5 | 12.7±1.3 | 13.3±1.5 | 13±0.3 | 13.7±0.5 | 14.3±0.7 | 0.60 | 0.08 | 0.01 |  |  |
| IBP-Mn6 | 18.1±0.5 | 19±0.4 | 19.5±0.6 | 17.4±0.9 | 18.4±1.2 | 19±1.0 | 17.4±0.7 | 18.2±0.7 | 18.2±0.8 | 18.3±0.6 | 19.1±0.8 | 19.1±0.8 | 0.76 | 0.01 | 0.00 |  |  |
| IBP-MF | 4.8±0.3 | 4.9±0.4 | 5.0±0.5 | 4.8±0.4 | 4.9±0.4 | 4.9±0.5 | 4.6±0.4 | 4.7±0.6 | 5.2±0.7 | 4.1±0.2 | 4.2±0.3 | 4.3±0.2 | 0.26 | 0.05 | 0.56 |  |  |

Units in mm; T0 for initial stage; T1 for second stage three months after initiation of experiment; T2 for final stage six months after initiation of experiment.

significant when p < 0.05 by linear mixed model analysis.

*p** for comparison of groups between group I, II and III; *p†* for comparison of saline- and BTX-treated side; *p‡* for comparison of time-related changes between T0, T1 and T2

Details can be seen in association with Figure 1B and 3 and Table S3.
