# Supplementary material for: Mandibular Vertical Growth Deficiency After Botulinum-Induced Hypotrophy of Masticatory Closing Muscles in Juvenile Nonhuman Primates
Source: Front Physiol. 2019 Apr 26;10:496. doi: 10.3389/fphys.2019.00496 (PMC6497797; doi:10.3389/fphys.2019.00496)
Supplement: TABLE S7 — Mandibular length measurements. [file Table_7.docx]

**Table S7. Mandibular length measurements.**

|  | Group I (control) | | | Group II (unilateral) | | | Group II (unilateral) | | | Group III (bilateral) | | | *p** | *p†* | *p‡* |  |  |
| --- | --- | --- | --- | --- | --- | --- | --- | --- | --- | --- | --- | --- | --- | --- | --- | --- | --- |
|  |  |  |  | - control side | | | - BTX side | | |  |  |  |  |  |  |  |  |
|  | T0 | T1 | T2 | T0 | T1 | T2 | T0 | T1 | T2 | T0 | T1 | T2 |  |  |  |  |  |
| Id-Con | 62.8±1.0 | 65.4±0.7 | 67.3±0.9 | 61±3.1 | 62.7±3.6 | 64.5±3.8 | 60.9±3.5 | 62.7±3.7 | 64.3±3.9 | 67.1±2.8 | 68.3±3.1 | 70.3±3.5 | 0.41 | 0.77 | 0.87 |  |  |
| Id-Cor | 52.9±0.7 | 55.2±0.7 | 56.9±1.1 | 51.5±3.0 | 52.4±3.5 | 54.1±3.9 | 50.8±3.7 | 51.9±3.8 | 53.1±4.5 | 56.6±2.6 | 57.1±3.1 | 59.4±2.8 | 0.10 | 0.53 | 0.03 |  |  |
| Id-Go | 53.5±0.5 | 55.9±0.6 | 57.1±0.7 | 52.2±2.3 | 53.8±2.2 | 55.6±2.8 | 51.8±2.3 | 53.6±1.9 | 54.3±2.3 | 57.4±1.4 | 57.9±1.7 | 59.2±3.2 | 0.76 | 0.33 | 0.30 |  |  |
| Id-Go(p) | 57.2±0.8 | 60.2±0.4 | 61.6±0.4 | 55.4±2.3 | 57.6±2.2 | 59.3±2.6 | 55.3±2.6 | 56.6±2.0 | 57.6±2.3 | 60.5±1.8 | 61.4±2.1 | 63.5±2.9 | 0.63 | 0.05 | 0.31 |  |  |
| Id-RA | 40.2±0.5 | 41.5±0.6 | 42.4±0.7 | 39.1±1.6 | 40±1.7 | 41.3±18.5 | 38.9±2.2 | 40.4±1.8 | 41.3±2.0 | 42.1±0.7 | 43±0.3 | 44.4±1.2 | 0.99 | 0.83 | 0.99 |  |  |
| RA-RP | 19.2±0.8 | 20.2±0.5 | 20.8±0.4 | 18.8±0.5 | 19.8±0.9 | 20.2±0.8 | 18.7±0.2 | 18.4±0.4 | 18.3±0.3 | 20.8±1.4 | 20.9±2.0 | 21.2±2.1 | 0.47 | 0.01 | 0.58 |  |  |
| Con(med)-Con(lat) | 9.7±0.4 | 10.2±0.5 | 10.4±0.6 | 9.7±0.9 | 10.4±1.0 | 10.6±1.0 | 9.6±0.7 | 10±0.7 | 10.5±0.9 | 9.7±0.8 | 9.9±0.7 | 10.1±0.8 | 0.10 | 0.82 | 0.02 |  |  |

Units in mm; T0 for initial stage; T1 for second stage three months after initiation of experiment; T2 for final stage six months after initiation of experiment.

significant when p < 0.05 by linear mixed model analysis.

*p** for comparison of groups between group I, II and III; *p†* for comparison of saline- and BTX-treated side; *p‡* for comparison of time-related changes between T0, T1 and T2

Details can be seen in association with Figure 1B and 3 and Table S4.
