# Supplementary material for: Mandibular Vertical Growth Deficiency After Botulinum-Induced Hypotrophy of Masticatory Closing Muscles in Juvenile Nonhuman Primates
Source: Front Physiol. 2019 Apr 26;10:496. doi: 10.3389/fphys.2019.00496 (PMC6497797; doi:10.3389/fphys.2019.00496)
Supplement: TABLE S8 — Mandibular width measurements. [file Table_8.docx]

Table S8. Mandibular width measurements.

|  | Group I (control) | | | Group II (unilateral) | | | Group III (bilateral) | | | *p‡* | *p** | Bonferroni^†^ |
| --- | --- | --- | --- | --- | --- | --- | --- | --- | --- | --- | --- | --- |
|  | T1 | T2 | T3 | T1 | T2 | T3 | T1 | T2 | T3 |  |  |  |
| Con-Con | 47.9±1.1 | 48.7±1.1 | 49.5±1.4 | 47±1.5 | 48.1±1.7 | 48.9±1.8 | 50.6±3.1 | 50.8±2.6 | 50.4±2.8 | 0.26 | <0.0001 | c>b, u>b |
| Cor-Cor | 50.5±1.3 | 51.8±1.9 | 52.4±2.1 | 49.5±0.9 | 50.4±1.1 | 51±1.2 | 52.7±2.3 | 53±1.8 | 53.4±1.4 | 0.19 | 0.07 |  |
| Go-Go | 32.2±1.9 | 33±2.0 | 33.8±1.6 | 30±1.3 | 30.7±1.0 | 31.2±1.2 | 33.5±3.5 | 33.4±2.9 | 34.1±3.1 | 0.83 | 0.62 |  |
| IAF-IAF | 33.9±0.8 | 34.4±0.9 | 34.8±0.9 | 33.2±1.2 | 33.8±0.9 | 33.9±1.1 | 36±1.0 | 36.1±1.3 | 36.8±1.2 | 0.98 | 0.96 |  |
| MF-MF | 13.6±2.4 | 14.4±2.4 | 15±2.4 | 13.9±1.3 | 14.4±1.2 | 15±1.3 | 15.5±1.2 | 15.7±0.7 | 15.9±0.7 | 0.55 | 0.02 | c>b |
| Mn6-Mn6 | 22.8±0.4 | 23.2±0.8 | 24.6±0.7 | 22.3±1.0 | 22.9±1.2 | 22.9±1.1 | 24.1±1.1 | 24.2±1.1 | 25.6±0.9 | 0.05 | 0.10 |  |
| RA-RA | 35.2±0.9 | 35.5±0.0 | 35.8±0.1 | 36.3±1.0 | 36.9±0.8 | 37.3±1.0 | 37±1.1 | 37.9±0.7 | 37.9±0.9 | 0.03 | 0.61 |  |

Units in mm; T0 for initial stage; T1 for second stage three months after initiation of experiment; T2 for final stage six months after initiation of experiment.

significant when p < 0.05 by two-way ANOVA.

*p** for comparison of groups between group I, II and III; *p‡* for comparison of time-related changes between T0, T1 and T2

*†* for Bonferroni correction after multiple comparison analysis; c for group I (control); u for BTX side of group II (unilateral); b for group III (bilateral)

Details can be seen in association with Figure 1C and 4 and Table S3.
