# Supplementary material for: Mandibular Vertical Growth Deficiency After Botulinum-Induced Hypotrophy of Masticatory Closing Muscles in Juvenile Nonhuman Primates
Source: Front Physiol. 2019 Apr 26;10:496. doi: 10.3389/fphys.2019.00496 (PMC6497797; doi:10.3389/fphys.2019.00496)
Supplement: TABLE S9 — Mandibular angular measurements. [file Table_9.docx]

Table S9. Mandibular angular measurements.

|  | Group I (control) | | | Group II (unilateral) | | | Group II (unilateral) | | | Group III (bilateral) | | | *p** | *p†* | *p‡* |  |  |
| --- | --- | --- | --- | --- | --- | --- | --- | --- | --- | --- | --- | --- | --- | --- | --- | --- | --- |
|  |  |  |  | - control side | | | - BTX side | | |  |  |  |  |  |  |  |  |
|  | T0 | T1 | T2 | T0 | T1 | T2 | T0 | T1 | T2 | T0 | T1 | T2 |  |  |  |  |  |
| IBP/Con-Go (MSP) | 108.4±2.6 | 108.3±1.9 | 109.8±1.7 | 106.5±1.9 | 106.6±2.0 | 105.5±0.7 | 106.4±1.0 | 107.4±2.8 | 111.2±3.1 | 108.2±3.8 | 110.3±4.2 | 113.4±3.4 | 0.38 | 0.001 | 0.81 |  |  |
| IBP/Con-IAF (MSP) | 131.2±2.2 | 132±2.5 | 132.5±3.0 | 131.1±5.7 | 131±4.1 | 130.6±3.7 | 131.2±4.0 | 132±4.9 | 134.6±6.2 | 135.5±4.7 | 136.1±5.8 | 137.9±5.1 | 0.64 | 0.28 | 0.17 |  |  |
| IBP/Cor-IAF (MSP) | 86.3±2.5 | 86.2±2.9 | 87.1±2.1 | 83.4±2.9 | 82.8±3.8 | 82.7±4.9 | 82.2±3.1 | 81.6±3.8 | 81.2±4.5 | 81±1.9 | 80.4±1.7 | 82.1±4.2 | 0.57 | 0.88 | 0.26 |  |  |
| IBP/MnOccP (MSP) | 8.1±2.4 | 9.5±1.1 | 10±1.3 | 7±2.9 | 7.2±2.8 | 6.3±3.1 | 5.6±3.2 | 7.2±2.2 | 9.2±2.9 | 7.3±1.1 | 7.8±1.9 | 10.4±2.2 | 0.17 | 0.003 | 0.78 |  |  |
| Con-Go / MnOccP (MSP) | 100.3±1.2 | 98.8±1.2 | 99.4±1.3 | 99.5±3.8 | 99.4±3.3 | 99.2±2.7 | 100.9±4.0 | 100.2±3.9 | 102±4.2 | 101±3.9 | 102.4±4.4 | 103±4.4 | 0.76 | 0.27 | 0.09 |  |  |
| Con-IAF/Cor-IAF (MSP) | 41.6±3.3 | 41.6±3.1 | 41.6±3.4 | 41.3±5.0 | 42.8±6.4 | 42.3±5.4 | 43.4±3.6 | 44.3±4.6 | 45.2±5.1 | 45±5.6 | 45.9±6.1 | 45±6.3 | 0.70 | 0.70 | 0.27 |  |  |
| MSP/Con-Go (CP) | 16.4±3.4 | 15.7±3.3 | 15.5±3.3 | 18.4±1.4 | 17.6±1.6 | 17.5±1.6 | 17.9±1.9 | 18.3±2.5 | 18.6±2.9 | 18.3±2.6 | 17.6±2.7 | 16.7±1.7 | 0.70 | 0.71 | 0.27 |  |  |
| MSP/Con-IAF (CP) | 22±2.0 | 22±2.4 | 22.2±2.7 | 23±3.0 | 21.6±2.1 | 22.1±2.6 | 23±2.5 | 24.9±2.9 | 25.8±3.4 | 23.8±2.0 | 23.1±2.6 | 21.7±1.4 | 0.002 | 0.004 | 0.8 |  |  |
| MSP/Cor-IAF (CP) | 25±2.7 | 25.2±2.6 | 24.7±2.9 | 25.2±1.0 | 25.3±1.3 | 26±1.3 | 25.8±1.6 | 25.9±1.2 | 25±2.1 | 25.1±1.8 | 25.3±2.8 | 23.9±3.3 | 0.25 | 0.18 | 0.16 |  |  |
| MSP/Me-Go(i) (FHP) | 25.3±2.1 | 25.1±2.1 | 24.9±1.7 | 23.6±1.0 | 22.4±0.9 | 22.2±0.5 | 22.6±2.0 | 24±1.1 | 24.5±1.4 | 23.9±3.2 | 24.1±3.1 | 24.5±3.0 | 0.18 | 0.000 | 0.82 |  |  |
| MSP/Li-Con (FHP) | 24.2±1.1 | 23.5±0.8 | 22.9±1.0 | 24.5±1.9 | 23.2±1.3 | 23±1.1 | 23.4±1.6 | 23.9±1.6 | 23.2±1.8 | 23.3±0.6 | 22.8±0.8 | 21.9±0.8 | 0.26 | 0.06 | 0.8 |  |  |

Units in degree; T0 for initial stage; T1 for second stage three months after initiation of experiment; T2 for final stage six months after initiation of experiment.

significant when p < 0.05 by linear mixed model analysis.

*p** for comparison of groups between group I, II and III; *p†* for comparison of saline- and BTX-treated side; *p‡* for comparison of time-related changes between T0, T1 and T2

Details can be seen in association with Figure 1D-F and 5 and Table S3.
