# Supplementary material for: Mandibular Vertical Growth Deficiency After Botulinum-Induced Hypotrophy of Masticatory Closing Muscles in Juvenile Nonhuman Primates
Source: Front Physiol. 2019 Apr 26;10:496. doi: 10.3389/fphys.2019.00496 (PMC6497797; doi:10.3389/fphys.2019.00496)
Supplement: TABLE S10 — Mandibular volume and area measurements. [file Table_10.docx]

|  | Group I (control) | | Group II (unilateral) | | Group II (unilateral) | | Group III (bilateral) | | *p** | *p†* |
| --- | --- | --- | --- | --- | --- | --- | --- | --- | --- | --- |
|  |  |  | - control side | | - BTX side | |  |  |  |  |
|  | T0 | T2 | T0 | T2 | T0 | T2 | T0 | T2 |  |  |
| Mn volume | 4476.3±173.0 | 6178.6±210.3 | 4016.3±291.3 | 5259.6±480.0 | 4021±275.3 | 4970±274.0 | 5240.1±418.1 | 5671.4±142.2 | 0.004 | 0.01 |
| Mn  surface | 4879.3±127.3 | 4819.2±314.8 | 4620.6±308.5 | 4435.2±517.9 | 4585.2±205.1 | 4397.4±490.9 | 5043.1±209.0 | 5760.3±553.4 | 0.89 | 0.01 |
| Mn ramus sectional area | 55.5±4.3 | 74.5±2.3 | 55.0±7.3 | 72.8±5.7 | 55.9±4.3 | 64.9±3.6 | 66.6±7.5 | 64.6±4.2 | 0.07 | 0.02 |
| Mn body  sectional area | 86.3±7.5 | 122.1±13.9 | 78.1±8.6 | 102.6±11.7 | 76.2±12.5 | 91.2±11 | 100.8±4.4 | 105.1±9.8 | 0.05 | 0.26 |
| Temporal muscle | 228.9±17.8 | 256±18.0 | 189.5±18.0 | 231.7±39.8 | 192.1±17.0 | 157.8±18.0 | 234.5±32.6 | 171.1±42.7 | 0.16 | 0.000 |
| Masseter muscle | 189.8±19.7 | 190.7±19.7 | 151.3±21.6 | 194.1±61.7 | 156.3±33.7 | 100±41.0 | 186.5±18.6 | 133.6±21.3 | 0.18 | 0.000 |
| Medial muscle | 82.8±6.4 | 79.4±6.4 | 77.1±8.7 | 83.2±12.2 | 78.8±11.0 | 44.7±10.3 | 103.1±17.9 | 86.2±33.9 | 0.27 | 0.003 |

Table S10. Mandibular volume and area measurements.

Units in mm^3^ for volume and in mm^2^ for surface and cross-sectional area; T0 for initial stage; T2 for final stage six months after initiation of experiment.

significant when p < 0.05 by linear mixed model analysis.

*p** for comparison of groups between group I, II and III; *p†* for comparison of saline- and BTX-treated side.

Details can be seen in association with Figure 1G-I, 2I-L, and 5 and Table S3.
